# Combination with antimicrobial peptide lyses improves loop-mediated isothermal amplification based method for *Chlamydia trachomatis* detection directly in urine sample

**DOI:** 10.1186/s12879-016-1674-0

**Published:** 2016-07-13

**Authors:** Jekaterina Jevtuševskaja, Julia Uusna, Liis Andresen, Katrin Krõlov, Made Laanpere, Tiia Grellier, Indrek Tulp, Ülo Langel

**Affiliations:** University of Tartu, Institute of Technology Laboratory of Molecular Biotechnology, Nooruse 1, Tartu, 50411 Estonia; SelfD Technologie GmbH, Leipzig, Germany; The Tartu University Hospital’s Women’s Clinic and Tartu Sexual Health Clinic, Tartu, Estonia; University of Tartu, Institute of Chemistry, Tartu, Estonia; Stockholm University, Stockholm, Sweden

**Keywords:** Loop-mediated isothermal amplification, Point-of-care assay, *Chlamydia trachomatis*, Pathogen detection in crude urine, Diagnostics of sexually transmitted diseases

## Abstract

**Background:**

*Chlamydia trachomatis* is an obligate intracellular human pathogen and is the most common cause of sexually transmitted diseases affecting both men and women. The pathogen can cause prostatitis and epididymitis in men. In women, cervicitis, pelvic inflammatory disease, ectopic pregnancy and acute or chronic pelvic pain are frequent complications. More than half of *C. trachomatis-*positive patients have minimal or no symptoms, providing an ongoing reservoir for the infection. The lack of sensitive large-scale applicable point- of- care (POC) tests for *C. trachomatis* detection makes it difficult to diagnose chlamydia infection efficiently in resource-limited environments.

**Methods:**

A rapid and sensitive assay based on loop-mediated isothermal amplification method (LAMP) was combined with antimicrobial peptide lysis, which is able to detect at least 7 *C. trachomatis* pathogens per reaction directly from urine samples.

**Results:**

Our study comprising 91 first-void urine samples showed that specificity of the assay is 100 % and sensitivity 73 % when using antimicrobial peptide lysis mix. Additionally we demonstrate that our assay does not give any cross-reactivity with 30 pathogen’s DNA potentially present in the urine samples. Furthermore, the assay’s novel approach does not require purification or extraction of DNA from clinical sample prior to amplification, so the need for specialized equipment is eliminated.

**Conclusions:**

The whole procedure is significantly less laborious, less time-consuming and consequently less expensive for early detection and identification of infectious disease. *C. trachomatis* specific LAMP assay is relatively simple to perform and could therefore be applied in numerous POC settings.

**Electronic supplementary material:**

The online version of this article (doi:10.1186/s12879-016-1674-0) contains supplementary material, which is available to authorized users.

## Background

*Chlamydia trachomatis* is a widespread sexually transmitted obligatory intracellular human pathogen, most often prevalent in adolescents and young adults of age 15–25 who have new or multiple sexual partners. The pathogen can also be passed from an infected mother to her baby during vaginal childbirth [1]. In men *Chlamydia trachomatis* usually cause prostatitis and epididymitis and is mostly symptomatic causing a mild to moderate, clear to white urethral discharge, burning sensation during urination or dysuria [2]. Patients infected with this pathogen have often minimal or no symptoms which impede the diagnosis. So it is very important to identify *C. trachomatis* in the early stage of infection and start immediate treatment as soon as possible to prevent the development of further long-term complications and decrease the chance of getting other infections such as *N. gonorrhoeae* or *Human immunodeficiency virus* [3].

The current gold standards for *Chlamydia trachomatis* clinical diagnostics is widely used PCR-based techniques (Abbot Real Time CT/NG assay; Roche Cobas Amplicor CT/NG assay eg) which is highly specific (amplify the specific DNA region of the multicopy cryptic plasmid) and sensitive but is time- consuming, requires complicated and expensive laboratory apparatus and need to prior extraction of genetic material from the sample. As an alternative different simple easy-to-use immunnoassay-based POC tests for detection *Chlamydia trachomatis* have been developed to reduce operating time, high-dependency, postoperative care time, numbers of outpatient clinic visits and ensure optimal use of professional time. In spite of the fact that most enzymatic POC tests have a high specificity up to 99 % they all have alarmingly poor sensitivity 0–30 % depending on test in comparison to nucleic acid amplification based techniques [4–9]. Limited sensitivity is a critical barrier to effective treatment and control of infectious diseases suggesting that there is still clear need for more sensitive and cost effective *C. trachomatis* diagnostic platforms.

As an alternative to the PCR based diagnostics, different nucleic acid amplification assays were developed allowing detection of pathogens in laboratories with a limited resources and POC settings. Although, novel nucleic acid amplification based tests are highly sensitive (91–100 %) [10, 11] they are susceptible to various biological components present in the sample, and thus do not allow the detection of pathogenic targets directly from crude extracts [12, 13]. Normally, unprocessed urine requires DNA extraction prior to amplification [14, 15] therefore several pathogen cell lysate preparation techniques can be suggested. Most of them however are complicated, time consuming and their application in POC settings require fully integrated automated platforms such as Cepheid GeneXpert assay, MAMEF assay, aQcare Chlamydia TRF kit [16–19].

Antimicrobial peptides (AMPs) have proven to be a very potent class of antimicrobial agents in vitro, being active in wide range of pathogens including Gram-positive and Gram-negative bacteria and fungi [20, 21]. Some peptides are able to efficiently lyse pathogen cells, releasing the DNA in the crude sample. The cell surface of the bacteria has a highly negative charge density suggesting that electrostatic interactions are responsible for their selectivity. The initial adhesion of the peptides to the cell membrane takes place with the helical axis parallel to the membrane surface [22–24] followed by insertion into the bilayer and disruption of the cell membrane [25, 26]. Therefore antimicrobial peptides could be potentially applied as fast and efficient sample preparation agents prior to isothermal amplification.

Loop-mediated isothermal amplification (LAMP) stands out to be a novel, highly sensitive and specific diagnostic tool because of the ease of performing and capability to diagnose a negligible amount of pathogen genetic material within an hour [27]. It has been reported that LAMP shows high tolerance to different inhibitors that are naturally present in various biological samples [28]. LAMP is carried out at a constant temperature (60–65 °C) and does not require a sophisticated and expensive thermal cycler. The amount of DNA product at the end of the LAMP reaction is considerably higher that than in PCR and can be simply visualized using metal ion indicators like calcein or such DNA biding dyes as SYBR green, ethidium bromide, picogreen, propidium iodide, hydroxy naphthol blue [29–34].

Here we combined *C. trachomatis* specific LAMP assay with antimicrobial peptide lysis for the rapid and sensitive pathogen detection from human urine which offers a good base for POC diagnostics platform. Application of antimicrobial peptides allows detection of *C. trachomatis* directly from urine sample, providing rapid, sensitive diagnosis with minimal need for training. Initial clinical analysis showed up to 73 % sensitivity and 100 % specificity of the developed *C. trachomatis* specific LAMP assay. The assay requires only 21 min and the product can be detected using lateral flow (LF) strips which is very beneficial for application in the point-of-care (POC) settings.

## Methods

### Design and selection of the primers

All *C. trachomatis* specific LAMP assay primer sets (Additional file 1: Table S1) were designed to target highly conserved region of the *C. trachomatis* cryptic plasmid within coding sequence 2 - CDS2 [13] using Basic Local Alignment Search Tool-BLAST (http://blast.ncbi.nlm.nih.gov) and LAMP Designer 1.10 software (PREMIER Biosoft USA). Additionally loop primers (LF/LB) were designed to accelerate amplification time and increase sensitivity. Primers were screened for their high sensitivity towards DNA target and for the absence of the non-specific background on LF strips. Oligonucleotides that contain sequence elements that promote secondary structures, hairpins or primer-primer interactions were avoided (using OligoAnalyser tool). Primers FIP and LF were 5′ labelled with biotin and BIP and LB primers with FAM to allow product detection on the LF strips. Primers were obtained from Microsynth AG (Balgach, Switzerland).

### LAMP reaction

*C. trachomatis* specific LAMP reaction was carried out according to the protocol supplied by Eiken Chemical Co. Ltd [35] with reaction total volume 50 μl. Bst polymerase was replaced with Bsm polymerase (Thermo Fisher Scientific Inc. USA) and 1.2 μl of template DNA and 15 μl of ddH20 were used. For untreated samples 5 μl of urine sample and 11.2 μl of ddH20 were used. All LAMP reactions were performed at 63 °C for 21 min and analysed on the LF strips (AMODIA Bioservice GmbH) according to manufacturer’s protocol. In order to confirm *C. trachomatis* amplification product specificity the reaction product was cut with PmlI restrictase (Thermo Fisher Scientific Inc. USA). The amplification product was considered specific if the signal on the LF disappeared after treatment with restrictase.

### Quantitative LAMP reaction

For quantitative analysis 0.5 μl EvaGreen (Biotium) fluorescent and 0.3 μl ROX (Thermo Fisher Scientific Inc. USA) reference dyes were added into standard LAMP reaction prepared as described above. Primers without Biotin/FAM labelling were used and product was detected using Applied Biosystems 7900HT Real-Time PCR System. A standard curve was constructed using serial dilutions of BglII linearized pGL3-CDS2 plasmid [13] at concentrations from 10^8^- 10^4^ with a 4 parallels. qLAMP was performed at 63 °C with 40 cycles, each 1 min long, data reading after 30 s.

*C. trachomatis* strain UW-36/Cx genomic DNA (ATCC® VR-886D™) was obtained from ATCC (American Culture Collection) and CDS2 target copy number was determined by qPCR (Applied Biosystems) using pGL3-CDS2 plasmid and following primers: Fw 5′**-** CTCCTTGGAGCATTGTCTGG**-** 3′ and Rw 5′-CGGATGCGATGAACAGTTTG**-** 3′.

### Sample pre-treatment

Lysates used for LAMP reaction were prepared by treating 30 μl of clinical patient’s urine sample with 4 μl of 1 × antimicrobial peptide lysis mix (SelfD Technologie GmbH, Leipzig, Germany; commercial component) for 5 min according to the protocol supplied by SelfD Technologie GmbH. Each lysate contained 12 % (from final volume) of peptide lysis mix and subsequently 1.2 % of peptide lysis mix in each LAMP reaction. Heat-treated samples were prepared by pre-heating urine at 90 °C for 5 min and then cooled down on ice. 5 μl of each lysates (heated urine or treated with antimicrobial peptide lysis mix) were then applied for *C. trachomatis* specific LAMP amplification. Pooled urine was only used (*C. trachomatis* negative urine mix from 5 men and 5 women in equal volumes) for assay sensitivity/specificity analysis.

### Statistical analysis

Statistical analyses of *C. trachomatis* specific LAMP assay’s limit of detection were performed with XLSTAT, 95 % confidence interval (CI) was calculated using logistic regression model. Assay sensitivity and specificity was calculated using online tool from MedCalc (http://www.medcalc.org/calc/diagnostic_test.php) confidence interval for 95 %.

### Clinical specimen collection and storage

First-void morning urine samples were collected from 650 patients from 18 to 25 year old (316 females and 334 males) attending Sexual Health Clinique (Tartu, Estonia) from October 2013 to December 2014. Urine samples were self-collected into a clean polypropylene container (average sample volume of 25–35 ml) without preservative and led by patients to the Sexual Health Clinique on the same day. Criteria for patient enrolment in the study were recent change of the sexual partner, multiple sexual partners, unprotected sexual intercourse, STI of the partner, and symptoms of the sexually transmitted disease.

Urine samples were tested by Cobas® 4800 CT/NG Test (Roche) for the presence of *C. trachomatis* according to manufacturer’s instruction by the United Laboratories of the Tartu University Hospital regularly on the second day of the sample collection, no longer than 72 h after collection.

The 91 non-frozen urine samples selected randomly out of 650 clinical specimens were tested by *C. trachomatis* specific LAMP assay within 6 h after collection. Approval for the study was obtained from the Research Ethics Committee of the University of Tartu.

## Results

### Validation of *C. trachomatis* specific LAMP assay and sample pre-treatment procedure

**Fig. 1:**
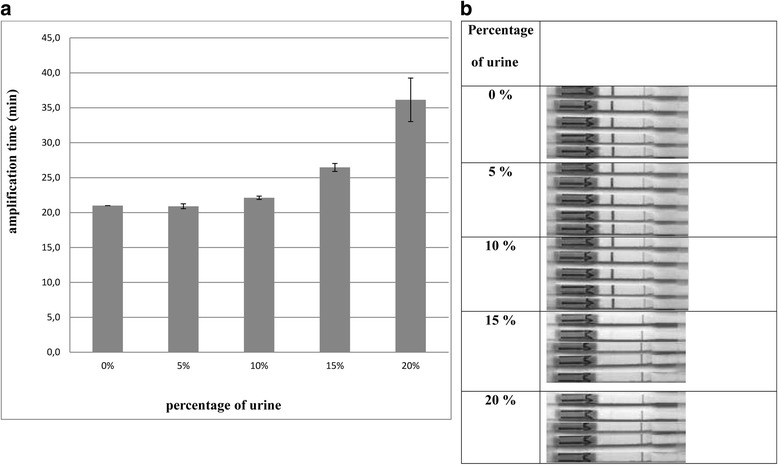
Urine tolerance of LAMP

Human urine contains variety of critical components that may inhibit the amplification entirely or only partially and by that prolong the amplification time. For instance, high concentration of urea in the sample (more than 50 mmol l^−1^) can lead even to the degradation of the polymerase [15, 36, 37]. In order to simplify pathogen detection procedure and make it more applicable for POC device settings, the DNA purification step prior the amplification was excluded. The amplification procedure has to take place directly in urine sample without previous concentration of the pathogen genetic material. Therefore, it is essential that developed amplification method has to tolerate as higher percentage of urine in the reaction mixture as possible allowing to increase the whole assay sensitivity as more targets are available for amplification. Our results demonstrated that reactions containing 15 % of urine did not give any signal when analysed on lateral flow strips, indicating that 21 min of amplification time was insufficient for target amplification. 5–10 % of urine in reaction mixture gave positive signal in all replicates (*n =* 5) (Fig. 1b). This observation was also confirmed by quantitative LAMP analysis where addition of 5–10 % urine did not affect amplification time (Fig. 1a). In the presence of 15–20 % of urine however, the amplification time should be prolonged up to 30 min indicating inhibitory effect of the urine components on LAMP reaction (Fig. 1a). Therefore based on this data, 10 % of urine was used as a sample material in all subsequent experiments. Our results showed that minimum reaction time required for product formation on LF strips was 21 min when as low as 100 copies of template per reaction were used in LAMP reaction containing not more than 10 % of crude urine sample (Additional file 1: Table S2). This enabled to reduce assay amplification procedure from recommended 1 h to 21 min without losing reaction sensitivity.

LAMP urine tolerance was tested in the presence of 0 %, 5 %, 10 %, 15 % and 20 % of pooled patient urine. Results were analyzed quantitatively (A) and using LF strips (B). For quantitative analysis, remaining amplification time was calculated as an average time compared to 0 % urine reaction at standard DNA dilutions 10^8^, 10^7^, 10^6^, 10^5^ and 10^4^ target copies per reaction. For LF strips detection, reaction was performed in the presence of 100 template copies in five parallels; two bands confirm the successful outcome of DNA amplification and a negative result is indicated by the presence of a single black band on the test strip.

**Fig. 2:**
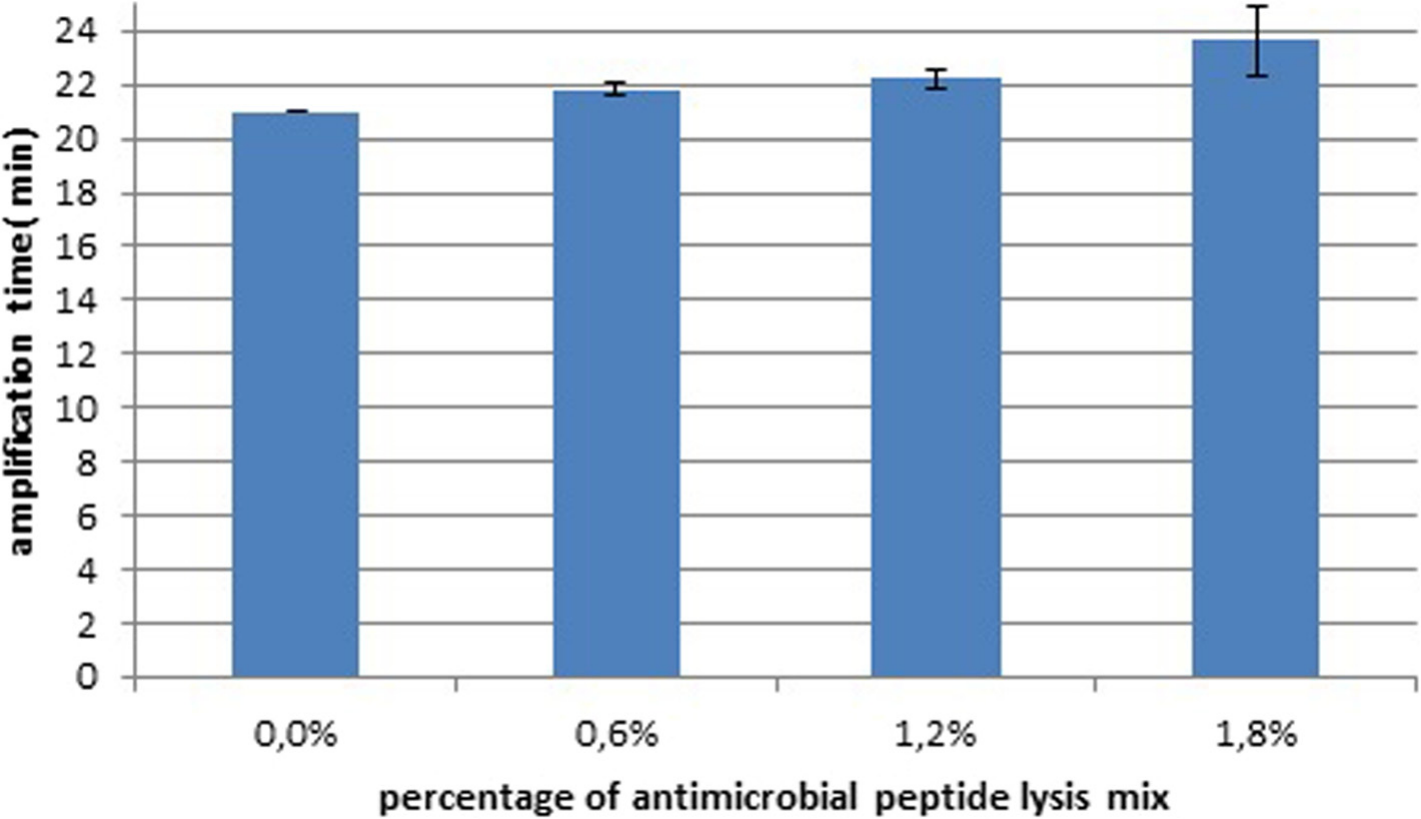
Tolerance of antimicrobial peptide lysis mix by LAMP

To eliminate the possibility that antimicrobial peptide lysis mix itself could inhibit LAMP reaction and find out the best concentration of lysis mix that could be applied in LAMP reaction, different lysis mix concentrations were tested. As a result addition of up to 1.2 % of antimicrobial peptide mix did not inhibit LAMP reaction as the amplification time was not altered. Based on this data up to 1.2 % of peptide lysis mix was used in LAMP assay without significant inhibition of amplification efficiency (Fig. 2).

Assay tolerance was tested in the presence of 0 %, 0.6 %, 1.2 %, and 1.8 % of 1 × antimicrobial peptide lysis mix (SelfD Technologie GmbH, Leipzig, Germany; commercial component). Results were analysed quantitatively. For quantitative analysis, remaining amplification time was calculated as an average time compared to 0 % antimicrobial peptide lysis mix reaction at standard DNA dilutions 10^8^, 10^7^, 10^6^, 10^5^ and 10^4^ target copies per reaction.

### Assessment of limit of detection (LOD) for *C. trachomatis* specific LAMP assay

Probit analysis showed that assay was sensitive enough to detect 25 plasmid copies per reaction in water (0.95 value) (Additional file 1: Figure S1). As developed assay does not contain DNA purification step it was important to estimate if 10 % of crude urine can affect the sensitivity. For that pooled urine samples were spiked with different amount of *C. trachomatis* genomic DNA. As a result, in the presence of 10 % urine sensitivity of LAMP assay reduced approximately three times and established LOD was 70 target copies per reaction (0.95 value) (Table 1).

**Table 1:** LOD determination for *C. trachomatis* specific LAMP assay^a^

	Copies per reaction	Lower bound 95 %	Upper bound 95 %
Limit of detection in water (0.95 value)	25	21	33
Limit of detection in pooled urine (0.95 value)	70	61	96

^a^For LOD determination in water 0, 4, 6, 12, 24, 48 and 120 plasmid copies were applied per reaction in 22 parallels each. For LOD determination in urine 0, 40, 50, 75, 100 and 200 plasmid copies were applied. Each reaction was performed in 20 parallels. Numbers of positive and negative signals visualized on LF strips were analysed by XLSTAT

These results are in good correlation with urine inhibition experiment, where addition of 10 % urine prolonged LAMP amplification time (Fig. 1a) and can be the reason why amplification activity was decreased approximately three times (Table 1).

### Determination of *C. trachomatis* specific LAMP assay specificity

Although LAMP primers were designed targeting pathogen specific region, their specificity had to be experimentally verified. *C. trachomatis* specific LAMP assay was further analysed for possible non-specific reactivity using excess amount of genomic DNA from human and 30 microorganisms potentially present in human urine (Additional file 1: Table S3). As a result *C. trachomatis* specific LAMP assay did not give cross-reactivity with any examined pathogen’s DNA and is highly specific in detection of chlamydia from human urine, fitting well for molecular diagnostic system.

### Clinical validation of *C. trachomatis* specific LAMP assay

To determine the prevalence of chlamydia in symptomatic and asymptomatic patients the urine samples subjected to clinical study (*N =* 650) were additionally evaluated. 157 out of 650 patients (24 %), had symptoms of STI and 493 patients (76 %) were asymptomatic. 51 % of the patients were males and 49 % females, with an average age of 22 years (18–25 years old). Chlamydia was diagnosed in 39 male patients and 47 female patients out of which only 12 (31 %) and 13 (28 %) had symptoms, respectively. Clinical data confirmed that *C. trachomatis* infection is mainly asymptomatic, which makes it difficult to diagnose and estimate the real prevalence in human population (Additional file 1: Table S4; Table S5).

*C. trachomatis* specific LAMP assay clinical sensitivity and specificity was validated in a study, comprising 91 freshly collected patient urine samples selected randomly from 650 clinical specimens. Although the number of specimens is not enough to provide high statistical power of the clinical study it was calculated to be minimal sample size to perform cost-effective assay validation with peptide lysis mix. First-void morning urine samples were self-collected by 56 male and 34 female patients and the overall prevalence of *C. trachomatis* among tested group was 12 %. Collected urine samples were subjected in parallel for *C. trachomatis* specific LAMP assay and Roche Cobas 4800 CT/NG test, a conventional technique for *C. trachomatis* detection. According to Roche Cobas 4800 CT/NG test 11 out of 91 had chlamydia infection. Two different sample pre-treatment strategies were tested and compared to untreated samples: heating at 90 °C for 5 min and incubation with peptide lysis mix for 5 min. From 80 *C. trachomatis* negative samples all tested negative in all treatments and no false-negatives were detected. Results showed that highest assay sensitivity (73 %) was obtained when using peptide/detergent lysis mix (out of 11 *C. trachomatis* positive urine samples 8 tested positive and 3 tested negative in the LAMP reaction) whereas pre-treatment with heat showed 64 % of assay sensitivity, and sensitivity dropped to 55 % when urine samples were left untreated (Table 2).

**Table 2:** *C. trachomatis* detection in 91 first-void urine samples with the Roche Cobas Amplicor CT assay and *C. trachomatis* specific LAMP assay

Fresh urine samples		*C. trachomatis* LAMP assay					
		Untreated urine^a^		Heat treated urine^b^		Peptide lysis mix pre-treated urine^c^	
		Positive	Negative	Positive	Negative	Positive	Negative
Roche Cobas Amplicor CT assay	Positive	6	5	7	4	8	3
	Negative	0	80	0	80	0	80
Sensitivity % [95 % CI]		55 % [23,38–83,25 %]		64 % [30, 79–89, 07 %]		73 % [39, 03–93, 98 %]	
Specificity % [95 % CI]		100 % [95, 5–100 %]		100 % [95, 5–100 %]		100 % [95, 5–100 %]	

^a^5 μl of fresh urine was added to LAMP reaction

^b^5 μl of urine was incubated at 90 °C for 5 min and after that added to LAMP reaction

^c^30 μl of urine was incubated with 4 μl (peptide lysis mix) for 5 min and after that 5 μl was taken out and added to LAMP reaction

These data clearly shows that pre-treatment of urine samples prior to amplification provide a better access of pathogen DNA material. All 80 chlamydia negative samples were detected as negative by developed LAMP assay, thus clinical specificity was estimated to 100 %.

## Discussion

Sexually transmitted infections (STIs), including highly prevalent *Chlamydia trachomatis*, remain an important focus area of the global public health [38]. Current *C. trachomatis* POC tests cannot offer the sensitivity comparable to laboratory nucleic acid amplification based techniques. Also concerns over the trueness of results, accuracy and general usefulness in non-emergency settings have slowed the adoption of these POC tests in general practice. Consequently successful detection and fast identification of chlamydia is necessary for early diagnosis and treatment of the disease. For that reason the need for simple and rapid on-site cost- efficient applicable *C. trachomatis* test which enables highly specific and sensitive pathogen detection remains.

This paper demonstrates for the first time, that the introduction of the antimicrobial peptide lysis step in the specific LAMP assay improves the detection of *C. trachomatis* directly in urine samples without prior extraction, purification and concentration of the pathogen genetic material. *C. trachomatis* cryptic plasmid has been chosen as a target for current diagnostic testing because it is present at up to 10 copies per genome [39]. Cryptic plasmid is relatively stable nucleic acid target being more resistant to nuclease damage than the genomic DNA. Selected CDS2 region shares high homology between different *C. trachomatis* serovars and is present in the Swedish mutant strains [13]. The use of six primers in LAMP reaction (F3, B3/FIP, BIP/LF, LB) provides not only a greater specificity than other amplification method but also accelerate the amplification time. Our data showed that novel *C. trachomatis* specific LAMP assay can detect at least 25 copies of the cryptic plasmid target in water. In urine, however, the sensitivity was 70 plasmid copies per reaction, probably due to potential inhibitors naturally present in crude sample material. Consequently, the newly developed LAMP assay is 100 % specific in detection of chlamydia. The whole assay takes less than 30 min, does not require additional equipment or trained personnel and the amplification product can be easily visualized by LF dipsticks and can be easily applied in the POC settings.

As simple and easy to apply sample pre-treatment method is one of the major criteria for the true self-diagnostic tests it is very important to find the best strategy for lysis of such challenging pathogen as chlamydia directly in crude urine. The lysis of the pathogen cells enables to liberate genetic material in such a way, that it would be suitable for the subsequent rapid amplification by LAMP method. To our knowledge only few reports related to detection of bacteria directly from biological sample using LAMP method are available [40, 41]. Current study shows for the first time novel and advantageous application of antimicrobial peptide mixture for the development of chlamydia specific LAMP assay directly in crude urine sample. Considering that majority of currently available POC tests have only 10–30 % of pathogen detection sensitivities, novel diagnostic approaches are required [42]. Our novel strategy successfully combined LAMP amplification with fast and simple sample preparation technique applying them directly in clinical samples. As a result the overall sensitivity of the novel *C. trachomatis* specific LAMP assay increased from 55 % (untreated, crude urine samples) up to 73 % when using antimicrobial peptide lysis mix and 64 % when samples were heat pre-treated prior addition into the amplification reaction. Thus by optimizing sample pre-treatment (for example urine sample enrichment) sensitivity of LAMP assay could be further increased to the levels comparable with commercially available nucleic acid amplification assays.

Our data shows that the application of antimicrobial peptide lysis mix provides promising sample pre-treatment strategy that suits all above mentioned requirements. Therefore there is no need to prior DNA extraction and purification, like in nucleic acid amplification -based chlamydia detection assays, which makes the whole detection less laborious and time consuming. Such features as increased sensitivity, controllability, simplicity, rapid response time and high specificity of pathogen detection compared to different POC immunoassays make antimicrobial peptide lysis combined *C. trachomatis* specific LAMP assay appealing for the application as reliable molecular diagnostic tool compatible for POC settings.

## Conclusions

We have developed for the first time simple, rapid and sensitive LAMP assay combined with antimicrobial peptide lysis mix for the specific detection of *C. trachomatis* directly from crude urine samples. *C. trachomatis* specific LAMP assay has great potential for the application in numerous POC settings.

## Abbreviations

CT, Chlamydia trachomatis; FAM - 6-carboxyfluorescein; NAAT, nucleic acid amplification technique; LAMP - loop-mediated isothermal amplification; POC, point-of-care; PCR, polymerase chain reaction; bp, base pair

## Additional file

Additional file 1: Table S1. LAMP primer set selected for *C. trachomatis* specific detection. **Table S2.** *C. trachomatis* specific LAMP assay amplification time. **Figure S1.** Probit curve for *C. trachomatis* -LAMP assay LOD in water (closed circle) and pooled urine (closed triangle) with lower and upper bounds 95 % in water and urine as described in lower panel of the image. **Table S3.** List of organisms used to determine specificity of the *C. trachomatis* LAMP assay. **Table S4.** Clinical characteristics of the studied group (*N =* 650). **Table S5.** Data for the patients with co-infection with NG. Additional information for the manuscript “Efficient and Rapid Loop-Mediated Isothermal Amplification Based Method for *Chlamydia trachomatis* Detection Directly from Urine”. (DOCX 59 kb)
