# Supplementary material for: Combination with antimicrobial peptide lyses improves loop-mediated isothermal amplification based method for Chlamydia trachomatis detection directly in urine sample
Source: BMC Infect Dis. 2016 Jul 13;16:329. doi: 10.1186/s12879-016-1674-0 (PMC4944247; doi:10.1186/s12879-016-1674-0)
Supplement: Additional file 1: Table S1. — LAMP primer set selected for C. trachomatis specific detection. Table S2. C. trachomatis specific LAMP assay amplification time. Figure S1. Probit curve for C. trachomatis -LAMP assay LOD in water (closed circle) and pooled urine (closed triangle) with lower and upper bounds 95 % in water and urine as described in lower panel of the image. Table S3. List of organisms used to determine specificity of the C. trachomatis LAMP assay. Table S4. Clinical characteristics of the studied group (N = 650). Table S5. Data for the patients with co-infection with NG. Additional information for the manuscript “Efficient and Rapid Loop-Mediated Isothermal Amplification Based Method for Chlamydia trachomatis Detection Directly from Urine”. (DOCX 59 kb) [file 12879_2016_1674_MOESM1_ESM.docx]

**Efficient and Rapid Loop-Mediated Isothermal Amplification Based Method for *Chlamydia trachomatis* Detection Directly from Urine**

**Supplementary Material**

**S1 Table.** LAMP primer set selected for *C. trachomatis* specific detection

| **Name** | **Sequence** |
| --- | --- |
| **F3** | 5’ AAT ATC ATC TTT GCG GTT GC 3’ |
| **B3** | 5’ TCT ACA AGA GTA CAT CGG TCA 3’ |
| **FIP** | 5’ Biotin-TCG AGC AAC CGC TGT GAC GAC CTT CAT TAT GTC GGA GTC 3’ |
| **BIP** | 5’ FAM-GCA GCT TGT AGT CCT GCT TGA GTC TTC GTA ACT CGC TCC 3’ |
| **LF** | 5’ Biotin-TAC AAA CGC CTA GGG TGC 3’ |
| **LB** | 5’ FAM-CGG GCG ATT TGC CTT AAC 3’ |

**S2 Table.** *C. trachomatis* specific LAMP assay amplification time

| **Reaction time (min)** | **Determination of percentage of positives*** |
| --- | --- |
| 15 | 20 % |
| 18 | 60 % |
| 21 | 100 % |
| 24 | 100 % |
| 27 | 100 % |

- Target copy number, detection on LF strips


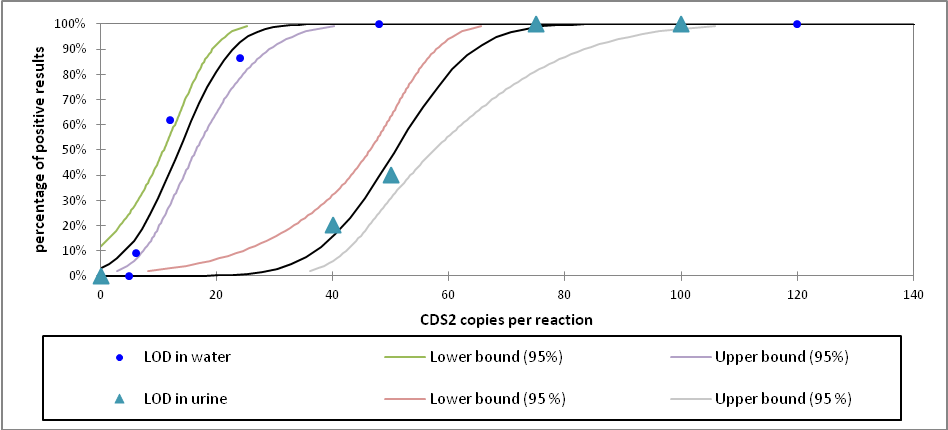


**S1 Fig.** Probit curve for *C. trachomatis* -LAMP assay LOD in water and pooled urine

Probit analysis of *C. trachomatis* specific LAMP assay´s limit of detection was performed in water and pooled urine. Statistical analysis was made with XLSTAT, 95 % confidence interval (CI) was calculated using logistic regression model. For LOD determination in water 0, 4, 6, 12, 24, 48 and 120 plasmid copies were applied per reaction in 22 parallels each. For LOD determination in urine 0, 40, 50, 75, 100 and 200 plasmid copies were applied. Each reaction was performed in 20 parallels. Numbers of positive and negative signals visualized on LF strips.

**S3 Table.** List of organisms used to determine specificity of the *C. trachomatis* LAMP assay

| **Species** | **Amount of DNA used per reaction** |
| --- | --- |
| **Mammalian** |  |
| - *Homo sapiens sapiens* | 1 ng |
| **Protozoa** |  |
| - *Trichomonas vaginalis* | 1 ng |
| **Fungi** |  |
| - *Candida albicans,* | 1 ng |
| - *Saccaharomyces cerevisiae* | 1 ng |
| **Bacteria** |  |
| *Escherichia coli* | 24 pg, 100 pg |
| *Neisserea gonorrhoeae* | 24 pg, 100 pg |
| *Mycoplasma genitalium* | 24 pg, 100 pg |
| *Mycoplasma pneumoniae* | 24 pg, 100 pg |
| *Ureaplasma urealyticum* | 24 pg, 100 pg |
| *Mycoplasma hominis* | 24 pg, 100 pg |
| *Lactobacillus iners* | 24 pg, 100 pg |
| *Lactobacillus crispatus* | 24 pg, 100 pg |
| *Corynebacterium urealyticum* | 24 pg, 100 pg |
| *Prevotella bivia* | 24 pg, 100 pg |
| *Gardnerella vaginalis* | 24 pg, 100 pg |
| *Veillonella parvula* | 24 pg, 100 pg |
| *Enterococcus faecalis* | 24 pg,- 100 pg |
| *Proteus mirabilis* | 24 pg, 100 pg |
| *Klebsiella pneumoniae* | 24 pg, 100 pg |
| *Klebsiella oxytoca* | 24 pg, 100 pg |
| *Enterobacter cloacae subsp. cloacae* | 24 pg, 100 pg |
| *Citrobacter freundii,* | 24 pg, 100 pg |
| *Staphylococcus saprophyticus subsp. saprophyticus* | 24 pg, 100 pg |
| *Streptococcus agalactiae* | 24 pg, 100 pg |
| *Staphylococcus epidermidis* | 24 pg, 100 pg |
| *Staphylococcus aureus subsp. aureus* | 24 pg, 100 pg |
| **Viruses** |  |
| *Herpes simplex 1* | 24 pg, 100 pg |
| *Herpes simplex 2* | 24 pg, 100 pg |
| *Human papilloma virus 16* | 24 pg, 100 pg |
| *Human papilloma virus 18* | 24 pg, 100 pg |

**S4 Table.** Clinical characteristics of the studied group (N=650)

| **Gender** | **N (%)** | **Mean Age** | **SYM*** | **ASYM*** | **CT prevalence in the clinical study** | **SYM** | **ASYM** | **CT negative** | **SYM** | **ASYM** |
| --- | --- | --- | --- | --- | --- | --- | --- | --- | --- | --- |
| **Male** | 334 (51%) | 22 | 70  (21%) | 264  (79%) | 39 | 12 (31%) | 27 (69%) | 295 | 57 (19%) | 238 (81%) |
| **Female** | 316 (49%) | 22 | 87  (27.5%) | 229 (72.5%) | 47 | 13 (28%) | 34 (72%) | 269 | 74 (27.5%) | 195 (72.5%) |

* Patient info analysis presents gender distribution, number and percentage of the *C. trachomatis* (CT) positive patients with symptoms (SYM) and without symptoms (ASYM).

**S5 Table**. Data for the patients with co-infection with NG

| **Gender** | **CT&NG**  **„+“** | **SYM** | **ASYM** | **CT”-”&NG„+“** | **SYM** | **ASYM** |
| --- | --- | --- | --- | --- | --- | --- |
| **Male** | 4 | 3 | 1 | 3 | 0 | 3 |
| **Female** | 3 | 3 | 0 | 6 | 1 | 5 |

* Gender and number of *C. trachomatis* and *N. gonorrhoeae* co-infected patients (CT&NG “+”), and *C. trachomatis* negative and *N. gonorrhoeae* infected patients (CT”-”&NG „+“) with symptoms (SYM) and without (ASYM).
